# Revealing momentum-dependent electron–phonon and phonon–phonon coupling in complex materials with ultrafast electron diffuse scattering

**DOI:** 10.1557/s43577-021-00156-7

**Published:** 2021-08-09

**Authors:** Hermann A. Dürr, Ralph Ernstorfer, Bradley J. Siwick

**Affiliations:** 1 Department of Physics and Astronomy, Uppsala University, P.O. Box 516, 75120 Uppsala, Sweden; 2 Fritz-Haber-Institut der Max-Planck-Gesellschaft, Faradayweg 4-6, 14195 Berlin, Germany; 3 Centre for the Physics of Materials, McGill University, 801 Sherbrooke St. W, Montreal, Canada

**Keywords:** Electron–phonon interactions, Quantum materials, Scanning transmission electron microscopy (STEM), Thermal conductivity, Laser-induced reaction, Time-resolved scattering

## Abstract

**Abstract:**

Despite their fundamental role in determining many important properties of materials, detailed momentum-dependent information on the strength of electron–phonon and phonon–phonon coupling across the entire Brillouin zone has remained elusive. Ultrafast electron diffuse scattering (UEDS) is a recently developed technique that is making a significant contribution to these questions. Here, we describe both the UEDS methodology and the information content of ultrafast, photoinduced changes in phonon-diffuse scattering from single-crystal materials. We present results obtained from Ni, WSe_2_, and TiSe_2_, materials that are characterized by a complex interplay between electronic (charge, spin) and lattice degrees of freedom. We demonstrate the power of this technique by unraveling carrier–phonon and phonon–phonon interactions in both momentum and time and following nonequilibrium phonon dynamics in detail on ultrafast time scales. By combining *ab initio* calculations with ultrafast diffuse electron scattering, insights into electronic and magnetic dynamics that impact UEDS indirectly can also be obtained.

**Graphic Abstract:**

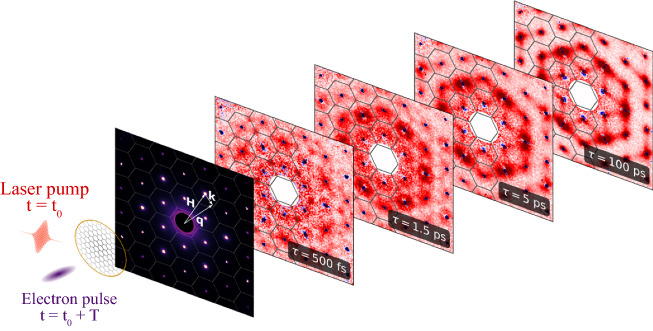

## Introduction

Elementary excitations and their mutual couplings form the fundamental basis of our understanding of diverse phenomena in materials. The interactions between collective excitations of the lattice system (phonons) and charge carriers, specifically, are known to lead to superconductivity, charge-density waves (CDW), multiferroicity, and soft-mode phase transitions.^1^ These carrier–phonon interactions are also central to our understanding of electrical transport, heat transport, and energy-conversion processes in photovoltaics^2^ and thermoelectrics.^3^ Phonons can themselves be intimately mixed in to the very nature of more complex elementary excitations, as they are in polarons or polaritons, or intertwined with electronic, spin, or orbital degrees of freedom, as it now seems is the case for the emergent phases of many strongly correlated systems that exhibit complex phase diagrams like high-*T*_c_ superconductors.^4^

Highly anisotropic (momentum-dependent) electron–phonon coupling (EPC) has been identified as a key feature of superconductivity in MgB_2_.^5^ It is also intertwined with electron correlations in the iron-based superconductor FeSe^6^ and has been shown to contribute to the selection of the electronic ordering vector in some charge-density wave materials, including ErTe_3_^7^ and NbSe_2_.^8^ Spin-dependent EPC plays an important role in nonequilibrium angular momentum exchange between electrons and lattice^9^ during ultrafast, laser-induced quenching of ferromagnetic order.^10^ On the other hand, phonon**–**phonon coupling (PPC) dictates the thermalization pathway of carrier/quasiparticle excitation energy, is a primary determinant of thermal conductivity in materials, and can contribute to stabilization of known phases in various materials (e.g., thermoelectrics such as SnSe and CsSnI_3_).

Our inability to fully characterize the nature of these elementary excitations and to quantify the strength of their momentum-dependent interactions has been one of the primary barriers to our understanding of these phenomena, particularly in complex anisotropic materials. Ultrafast pump-probe techniques provide an opportunity to study couplings between elementary excitations in a direct way. Photoexcitation can prepare a nonequilibrium distribution of quasiparticles or other selected modes whose subsequent relaxation dynamics and coupling to other degrees of freedom can be followed in the time domain. Ultrafast electron scattering has proven to be extremely powerful in this regard, due to the direct connection between the scattering observables and the structure and dynamics of the lattice.

In this article, we highlight the potential of ultrafast electron diffuse scattering **(**UEDS) for three examples: The case of ferromagnetic Ni illustrates the influence of ultrafast electronic structure changes on the energy flow between electrons and phonons. We show how UEDS reveals momentum-dependent EPC and PPC for two-layered crystals, the semiconductor WSe_2_, and the semimetal TiSe_2_ featuring laser-induced phonon softening. We stress that these processes are initiated by nonequilibrium laser excitation and are therefore inaccessible to near-equilibrium techniques such as inelastic neutron scattering. The following section describes the basic ideas behind UEDS.

## Ultrafast electron diffraction and phonon-diffuse scattering

**Figure 1:**
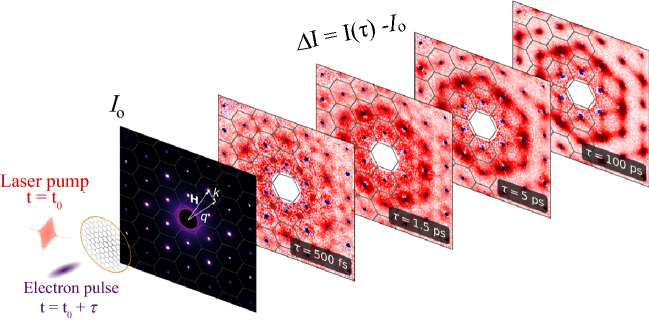
Ultrafast electron scattering experiment. The material to be studied is first “pumped” with a femtosecond laser pulse whose frequency is suitably chosen to selectively excite specific degrees of freedom (e.g., carriers or phonons). After a precisely controlled time delay, τ, an ultrashort electron pulse is scattered off the laser excited material and forms a scattering pattern. The pump-induced changes to scattering intensity, Δ*I*(***q***,*τ*) = *I*(***q***,τ) − *I*_o_(***q***), where *I*_o_ is the intensity before photoexcitation, can be determined at all scattering vectors, ***q***. Encoded in the scattering pattern is a “snapshot” of the instantaneous crystal structure through the time-dependent intensity of Bragg peaks (blue). Changes in phonon mode amplitude at all momenta throughout the Brillouin zone (shown as black lines) are encoded in the time-dependent phonon-diffuse scattering intensity between the Bragg peaks (red).^24^

UEDS is based on a pump–probe technique, whereby a femtosecond laser-pulse “pumps” (or excites) the sample and an ultrashort electron pulse “probes” the subsequent dynamics in one of several of ways (**Figure** **1**). First, it is now well established that detailed information on atomic and charge reorganization can be followed with femtosecond time resolution using ultrafast electron diffraction (UED).^11–14^ Via UED, it is now possible to watch the coherent, directed motions of atoms and the associated reorganization of electrons in materials on their natural time scales in response to optical excitation, enabling “molecular movies” of fundamental dynamics (other articles in this issue will describe such applications). Second, incoherent vibrations of the atoms following optical excitation can be directly measured through the time-dependent Debye–Waller effect by monitoring the intensity of Bragg scattering peaks.^15^ More recently, improvements in ultrafast electron sources^16–18^ have provided access to the small scattering signals outside of Bragg peaks. It is now possible to reliably make use of the information contained in the time-dependent diffuse scattering signals between the diffraction peaks of a UED pattern (Figure 1). It has been demonstrated that this ultrafast electron diffuse scattering (UEDS) can be used to determine the nonequilibrium occupancy of phonon modes across the entire Brillouin zone (BZ) in a single-crystalline material with femtosecond time resolution.^19–25^ This information can be used to determine the strength of the wavevector-dependent (or momentum-dependent) coupling between electrons and phonons and the strength of anharmonic coupling between phonons themselves. The information UEDS provides is analogous to time- and angle-resolved photoelectron spectroscopy (TR-ARPES),^26–28^ but for the phonon system rather than the electron system.

These capabilities are entirely due to the profound sensitivity of UEDS signals to nonequilibrium phonon dynamics and occupancies. The diffuse scattering from phonons, appearing between the Bragg peaks, is described by the following equations:^29,30^

1 $$I\left({\varvec{q}}\right)={\sum }_{j}\frac{{n}_{j}\left({\varvec{q}}\right)+1/2}{{\upomega }_{j}\left({\varvec{q}}\right)}{\left|{F}_{j}\left({\varvec{q}}\right)\right|}^{2},$$

with

2 $${\left|{F}_{j}\left({\varvec{q}}\right)\right|}^{2}={\left|{\sum }_{s}\mathit{exp}\left(-{W}_{s}\left(q\right)\right)\frac{{f}_{s}\left(q\right)}{\sqrt{{\upmu }_{s}}}\left({\varvec{q}}\cdot {\mathbf{e}}_{\mathrm{j},\mathrm{s},\mathbf{k}}\right)\right|}^{2}.$$

Equation (1) shows that $I\left({\varvec{q}}\right)$, the diffuse intensity at scattering vector ***q***, depends on the occupancy of phonon modes ${n}_{j}\left({\varvec{q}}\right)$ divided by the mode frequencies ${\upomega }_{j}\left({\varvec{q}}\right)$ summed over all phonon branches *j* (please see Figure 1 for a definition of the **k**-vectors). The intensity of scattering at ***q*** also depends on the one-phonon structure factor, *F*_*j*_*,* defined in Equation (2). *F*_*j*_(***q***) depends only on the polarization vectors **e**_j,s,**k**_ of each basis atom *s* for the phonon mode in branch *j* with momentum/wavevector ***k*** (where ***k*** depends on the position relative to the closest Bragg peak, **H**, according to ***q*** = **H** – ***k)***. Only phonon modes with wavevector ***k*** contribute to the scattering intensity at ***q***. *F*_*j*_(***q***) can be robustly computed using density functional theory methods.^25^ UEDS probes the time-dependent changes to ${I}\left({\varvec{q}}\right)$ that are induced by photoexcitation, and therefore directly measures changes in phonon amplitude ${n}_{j}\left({\varvec{q}}\right)/{\varvec{\upomega }}_{j}\left({\varvec{q}}\right)$ at all phonon wavevectors in the BZ. This includes phonon creation/annihilation in response to laser excitation at all wavevectors via ${n}_{j}\left({\varvec{q}}\right)$, as well as the possibility of measuring the renormalization of mode frequencies ${\upomega }_{j}\left({\varvec{q}}\right)$*.* We give examples of these novel observations from previous work next.

## Ultrafast nonequilibrium energy flow between electronic and lattice degrees of freedom in crystalline nickel

Ferromagnetic Ni has been the first material where in 1996 an unexpected subpicosecond quenching of the ferromagnetic order upon laser heating was discovered.^10^ Since then, this process has been a basic ingredient for a plethora of additional ultrafast optomagnetic phenomena.^31^ However, the direct observation of ultrafast energy transfer between laser-heated electrons and the lattice has remained enigmatic. Here we describe for this prototypical metallic ferromagnet how electron-lattice energy transfer is influence by nonequilibrium demagnetization.

**Figure 2:**
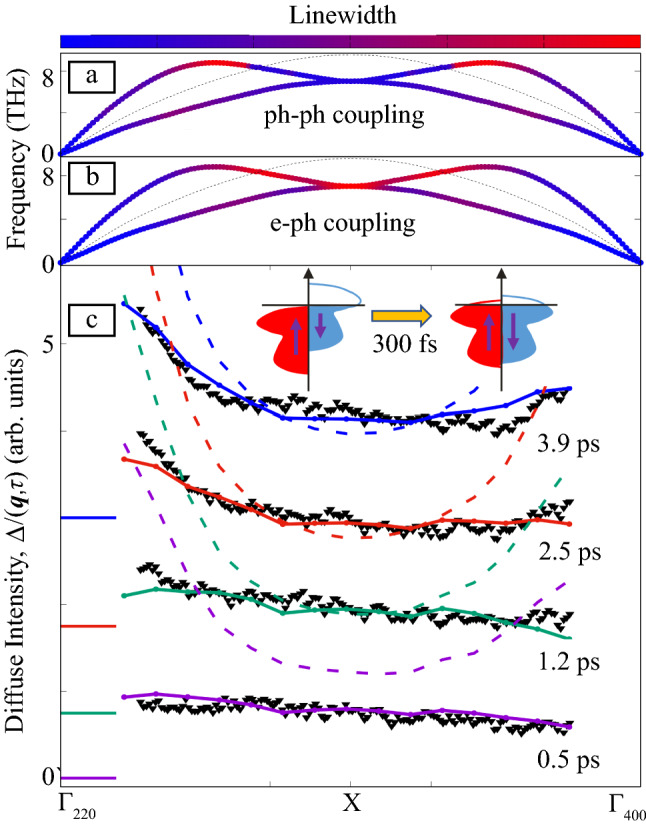
Electron–phonon energy transfer during a magnetic phase transition. Ultrafast demagnetization leads to characteristic changes in the spin-resolved electronic structure within 300 fs as illustrated in the inset. The process of energy transfer is governed by phonon–phonon coupling (PPC) (a), and electron–phonon coupling (EPC) (b), shown in the figure overlaid to the phonon frequency dispersions along a direction in reciprocal space. (c) Measured (symbols) and calculated (lines) diffuse scattering intensity, ∆*I*(***q***, *τ*), for the indicated times, *τ*, from a Ni film that undergoes a femtosecond laser-induced magnetic phase transition as described in the text from Reference 25. The solid (dashed) lines correspond to calculations without (with) a magnetic phase transition, respectively.

**Figure** **2** shows an example of measured and calculated Ni phonon properties for phonon wavevectors along the Γ-X-Γ high-symmetry line in reciprocal space. This can be probed by measuring the UEDS intensity between the (220) and (400) Bragg peaks.^25^ Figure 2a–b displays the calculated phonon dispersions along this line. The experimentally detectable phonon dispersions are overlaid by computed phonon linewidths due to PPC and EPC that ultimately determine the energy flow from laser-exited electrons to phonons. Figure 2b shows that the largest EPC takes place predominantly for the modes around the BZ edges (X points) at high momentum values. In contrast, the PPC shown in Figure 2a provides the largest linewidth contribution for the phonon modes with highest frequencies.

The resulting transient phonon populations are probed in Figure 2c for the indicated times following excitation with a femtosecond laser pulse. The individual time traces are offset vertically for clarity. The curves correspond to the laser-induced changes in the phonon population described by Equations (1 and 2). The wavevector ranges are limited approaching the Γ points due to appearance of Bragg peaks with intensities significantly larger than the diffuse scattering. The UEDS intensities display characteristic changes with time delay: The total amount of scattering increases and the intensity distribution shifts toward the Γ points. This is qualitatively expected for a thermalized phonon population.^19^ However, a quantitative description reveals that the transient phonon populations display significant nonequilibrium character for all times displayed in Figure 2.^25^ Lines in Figure 2c represent two calculated scenarios, transient phonon excitation for ferromagnetic Ni (dashed lines) and for a ferromagnetic to nonmagnetic phase transition (solid lines). It is evident that the purely ferromagnetic case does not reproduce the experimental observations. In this case there is a fast transfer of electronic energy into phonons resulting in a much higher mode occupation, and also a faster relaxation within the lattice, than experimentally observed at all pump-probe delay times. It is important to note that only one single global scaling factor has been applied to match experimental and theoretical diffuse scattering intensities, which demonstrates the predictive power the UEDS approach.

This demonstrates how ultrafast changes in magnetic order can influence lattice properties. Ni demagnetizes upon ultrafast laser excitation within several 100 fs.^10^ The collapsing exchange splitting on demagnetization changes the electronic structure of Ni.^26,28^ This situation is depicted schematically in the inset of Figure 2c. When the exchange splitting of spin-up (↑) electronic states (red) and spin-down (↓) states (blue) is reduced, the density of states around the Fermi level mainly responsible for EPC changes dramatically. In ferromagnetic Ni, the EPC is mainly caused by spin-down *d*-electrons close to the Fermi level. It is these spin-down states that are affected most by demagnetization. These time-dependent changes of the EPC strength can be accounted for by a reduction of its strength (solid lines in Figure 2c). This results in an excellent agreement with the measurements for all time delays and phonon modes.

**Figure 3:**
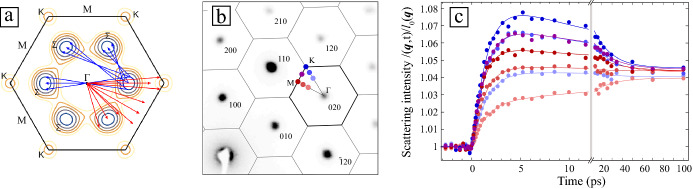
Ultrafast electron diffuse scattering of WSe_2_. (a) Illustration of electron intervalley scattering processes between Σ valleys of the conduction band (blue arrows) and the associated phonon wavevectors (red arrows). For instance, second-next Σ valleys are connected via M-point phonons. (b) Ultrafast electron diffuse scattering (UEDS) pattern of photoexcited WSe_2_. Hexagons indicate the Brillouin zone surrounding the Bragg spots. (c) The time-dependent diffuse scattering signal *I(****q****,τ)* (circles) for ***q***-values along high-symmetry lines as indicated in (b). Solids lines show biexponential fits of the data. Reproduced from reference.^22^

A further surprising result is the appearance of a backflow of energy from high-energy phonon modes to electrons at times larger than 1 ps. This process is caused by the maxima of EPC and PPC occurring at different wavevectors (see Figure 2a–b). Thus, a transient phonon population can survive near the X-point until the electrons have cooled down far enough to be reheated by energy transfer from phonons. Experimentally, it leads to a maximum of the X-point UEDS intensity for times around 1 ps, while for smaller wavevectors only a monotonous intensity increase is observed (see Figure 3 in Reference 25).

These results demonstrate a robust and straightforward way to disentangle the complex nonequilibrium interplay between electrons and phonons that can be extended to more complex materials. The energy exchange between electrons and lattice has an explicit dependence on the magnetic character of the system, and magnetization changes entails significant modification of the lattice dynamics. We expect significant implications also for the nonequilibrium electron and spin motion which can be investigated using, for instance, momentum-resolved detection of spin wave dynamics.^32^

## Revealing energy dissipation pathways in the semiconductor WSe_2_

The development of new materials and device concepts advancing device performance at reduced energy consumption is a persistent challenge in materials science. On the microscopic level, device functionality arises from controlled motion of electron wave packets in nonequilibrium states of crystals, typically semiconductors. Microscopic interactions lead to constant scattering of the wave packets in energy–momentum space with EPC being the governing dissipative channel^33^ and the key process for realizing low-dissipation devices. WSe_2_ in the 2H crystal structure is a layered transition-metal dichalcogenide semiconductor that can be viewed as a model compound to study these processes.

**Figure** **3** illustrates how UEDS provides momentum-resolved information on EPC for the case of optically excited WSe_2_. Optical excitation generates hot electrons accumulating in the lowest conduction band valleys at the six Σ points of the BZ,^34^ as indicated by the energy contours in Figure 3a. Energy dissipation occurs through the emission of phonons and eventually condenses the electrons at the conduction band minima.^22^ The electron-transport properties in the excited state depend on the nature of the electron–phonon scattering processes (e.g., if these are governed by intravalley or intervalley scattering between neighboring Σ valleys), as indicated by blue arrows. While TR-ARPES cannot distinguish these processes, the momentum distribution of the emerging phonon population reveals the phase space of the electron scattering processes. Figure 3b shows an UEDS image with the material’s hexagonal BZs indicated around the Bragg spots. According to Equation (1), the time- and momentum-dependent scattering intensity *I*(***q***,*τ*) reflects the evolution of the branch-integrated phonon population *n*(***q***). Figure 3c shows the build-up of phonon population at selected momenta in the outer part of the BZ.^22^ The pronounced rise of signal at the M (dark red) and K (dark blue) points reveals the strong contribution of intervalley scattering between second- and third-nearest Σ valleys, as these are connected by M and K valley phonons, respectively, as indicated by red arrows in Figure 3a. The initial phonon population is nonthermal, as evidenced by the differences in the relative scattering intensities at early times (see Figure 3c). Thermal distribution is established through phonon–phonon scattering within tens of picoseconds. The time scales retrieved from UEDS therefore provide benchmarks for first-principles calculations for both EPC and PPC. Due to the high scattering cross section of electrons, the approach can straightforwardly be extended to quantify exciton-phonon coupling^35^ in true 2D materials.

## Strong electron–phonon coupling and soft phonons in TiSe_2_

This section describes the increased complexity of EPC in the presence of soft phonon modes using TiSe_2_ as a model system. A layered transition-metal dichalcogenide,^36^ TiSe_2_ in the 1T crystal structure exhibits a rich phenomenology emerging from EPC.^37^ TiSe_2_ is an indirect semimetal at room temperature with electron pockets located at L points and a hole pocket at Γ.^38^ A commensurate CDW phase forms below ${T}_{c}\approx 190$ K that exhibits a $2\times 2\times 2$ superlattice reconstruction.^39^ This transition is preceded by the observable softening of the entire M-L transverse phonon branch over a temperature range greater than 150 K above ${T}_{c}$, suggesting that EPC could play an important role in the emergence of CDW order and the selection of the ordering vector.^40^ This softening has been investigated by both diffuse and inelastic x-ray scattering^41^ and static momentum-resolved electron energy-loss experiments.^42^ These works along with other studies point to the strong influence of electron–hole correlations that in turn may drive the CDW transition. This scenario is best understood as an exciton condensate predicted more than 50 years ago.^43^ In the Cu-intercalated species, Cu_*x*_Ti_1−*x*_Se_2_, CDW order is quenched, yielding a superconductor,^44^ which suggests a delicate relationship between the carrier concentration and the lattice stability.

**Figure 4:**
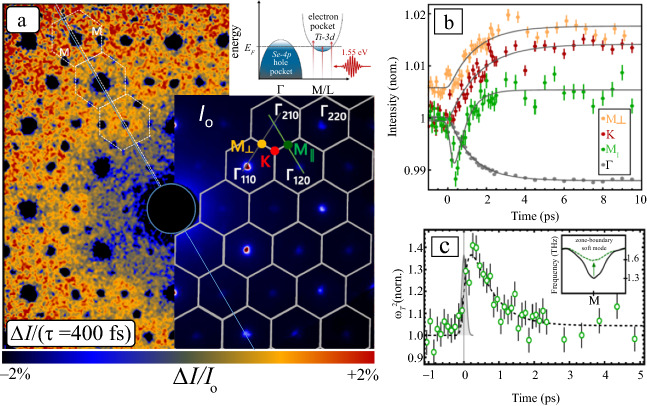
Ultrafast electron diffuse scattering in the normal phase (300 K) of TiSe_2_. (a) The laser pump (35 fs, 800 nm, 4 mJ/cm^2^) photo-dopes carriers into the electron pocket at the Fermi level as shown in the inset. Electron scattering intensity change, Δ*I*/*I*_o_, following photoexcitation at a pump-probe time delay of 400 fs. *I*_o_, the equilibrium (300 K) electron scattering pattern, is overlayed. Regions of decreasing intensity are only found at (1) M points where strong phonon-diffuse intensity from the soft mode appears at equilibrium and (2) Bragg peaks due to the transient Debye–Waller effect. (b) Transient diffuse intensity at several points in the Brillouin zone (BZ) indicated in (a). The pronounced dip in diffuse intensity at the M_1_ point (green symbols) is due to the renormalization (stiffening) of the zone-boundary soft-mode frequency following photoexcitation. At other BZ-boundary points (K, red symbols and M_⊥_, yellow symbols) there is mainly a monotonous increase, while the Γ-point (black symbols) shows a Debye–Waller-like decrease in intensity. (c) The inferred soft-mode frequency versus time following photoexcitation.^46^

UEDS measurements on TiSe_2_ in the normal phase at 300 K have revealed the fundamental mechanisms that underlie the observed softening of the zone-boundary transverse phonon branch along M-L of the BZ, which is associated with the three-dimensional CDW transition at lower temperatures. In these experiments, photoexcitation at 1.55 eV (800 nm) effectively “photo-dopes” additional carriers into the electron pockets near the Fermi level (see** Figure** **4**a inset). The response of the lattice to this photocarrier doping was followed by UEDS. Figure 4a shows the diffuse scattering intensity, ${I}\left({\varvec{q}}\right)$, at a pump-probe time delay of 400 fs. Immediately evident is the anticipated reduction in Bragg peak intensities at the ${{\Gamma}}$ points (BZ center) due to the Debye–Waller effect. In addition, however, a striking and surprising intensity decrease is found at certain BZ-boundary M points (two such points are indicated in Figure 4a). The only points showing this effect are those that also exhibit strong phonon-diffuse signal from the soft transverse zone-boundary phonon mode (previously described) at equilibrium. Figure 4b shows the complete time-dependence of the change in scattering intensity at M, K, and Γ points in the BZ (indicated in Figure 4a) following photoexcitation. These data revealed that the rise-time for diffuse scattering intensity from phonons increases at all points outside the selected M points of the BZ previously mentioned are in agreement with those shown at K and M_⊥_ within the reported uncertainties. Phonons of all wavevectors appear to be excited at the same rate. The most striking and unusual feature of these data, however, is the quasi-impulsive anisotropic suppression in diffuse intensity at specific M points (green symbols in Figure 4b). This feature was assigned to carrier-induced renormalization (stiffening) of the soft zone-boundary (M and L) transverse phonon mode frequency ${{{\upomega}}}_{T}\left({{q}}={M},{L}\right)$. The carrier-induced increase in frequency of this mode leads directly to a decrease in diffuse intensity at the M points as can be understood from Equation (1). This phenomenon can be distinguished from the heating of phonon modes throughout the BZ, which is observed as an increase in diffuse intensity (Figure 4b, red and yellow symbols) and occurs on an order of magnitude slower time scale than the observed stiffening.

Together, these data lead directly to the conclusion that the rate at which energy is exchanged between photo-doped carriers and phonons is not significantly enhanced for the soft zone-boundary phonon. By contrast, the phonon frequency renormalization associated with photoexcitation is strongly wavevector specific (i.e., only observed at BZ positions associated with the soft transverse phonon).

Photo-doping free carriers into the electron pocket of TiSe_2_ appears to selectively “decouple” the soft zone-boundary transverse phonon at M, thereby stiffening the lattice vibration. The change in frequency of this mode, ω_T_, can be directly determined from the UEDS signals, and is directly correlated with the free-carrier density in the electron pockets at M and L points (Figure 4c). The presence of free carriers in the electron pockets are intimately connected to the mode-softening/stiffening, suggesting that the primary microscopic mechanism is dielectric screening or photocarrier modification of the many-body electronic susceptibility.^45^

In striking contrast to graphite where the phonon modes that exhibit Kohn anomalies are also those into which electronic excitation energy flows most rapidly,^23,24^ in TiSe_2_ there is no evidence of such an anisotropy. From the perspective of the rate at which energy is transferred between free carriers and phonons, electron–phonon coupling is isotropic.

These results point to a highly anisotropic electronic susceptibility, strongly dependent on the free-carrier density in the electron pockets, as the dominant microscopic mechanism driving the temperature-dependent phonon softening observed in measurements of TiSe_2_ at equilibrium.

## Summary and outlook

Ultrafast electron diffuse scattering has emerged as a powerful tool for the investigation of phonon dynamics in crystals. By utilization of the phonon-symmetry dependence of the inelastic scattering process in addition to the intrinsic resolution of ultrafast scattering, transient phonon populations of nonequilibrium states can be resolved in time, momentum and phonon branches. Despite the lack of explicit energy resolution, UEDS provides the equivalent information on ultrafast phonon dynamics as time-resolved ARPES does for electrons. The method can be implemented with nonrelativistic tabletop as well as facility-based relativistic UED setups and promises new levels of microscopic insight to vibrational excitations and microscopic interactions in nanoscale quantum materials and heterostructures. Recent experimental advances demonstrated pulse length compressing using THz laser pulses^47,48^ as well as single-electron detection.^49^ This will significantly improve time resolution, dynamical range and ultimately also the momentum resolution of future UEDS experiments.
